# The effect of sensor-to-source distance on magnetic neuromuscular signals

**DOI:** 10.1038/s41598-025-06545-1

**Published:** 2025-06-20

**Authors:** Haodi Yang, Thomas Klotz, Leonardo Gizzi, Hongyu Lu, Gianpiero Monittola, Urs Schneider, Markus Siegel, Justus Marquetand

**Affiliations:** 1 Department of Neural Dynamics and Magnetoencephalography, Hertie-Institute for Clinical Brain Research, University of Tübingen, Otfried-Müller-Str.47, 72076 Tübingen, Germany; 2 MEG-Center, University of Tübingen, Tübingen, Germany; 3 Center for Integrative Neuroscience, University of Tübingen, Tübingen, Germany; 4 Institute for Modelling and Simulation of Biomechanical Systems, University of Stuttgart, Stuttgart, Germany; 5 Fraunhofer Institute for Manufacturing Engineering and Automation (IPA), Stuttgart, Germany; 6 Center for Mind/Brain Sciences (CIMeC), University of Trento, Rovereto, Italy; 7 Center for Bionic Intelligence Tübingen Stuttgart (BITS), Tübingen, Germany

**Keywords:** Signal decay, Simulation, Optically pumped magnetometer, magnetomyography, Amplitude, Frequency, Neuroscience, Neurology

## Abstract

Magnetomyography (MMG) can be used as a contactless modality to study the neuromuscular system. On the one hand, being contactless is a practical advantage as there is no need to prepare skin or attach electrodes as in electromyography (EMG). On the other hand, it is also a disadvantage because the magnetic field decays with increasing distance. However, the effect of sensor-to-source distance in MMG has not been systematically studied. Comparative in vivo and in silico experiments of the effect of sensor-to-source distance were performed. In vivo, muscle activity was recorded using simultaneous surface EMG and one triaxial optically pumped magnetometer (OPM). For the simulations, an established multiscale muscle model was used to predict how distance affects the signal-to-noise ratio (SNR) and the signal’s spectral content. Given an environmental noise level of 0.5–1 pT root-mean-square (RMS) from 10 to 350 Hz, it was impossible to robustly detect muscle activity of one finger flexor muscle beyond a distance of two centimeters using OPM technology. In silico experiments showed a high SNR between 8 and 29 for MMG at 0.5 cm distance. Increasing the distance increases the MMG’s median frequency content. The simulations uncovered that this is due to the effect of noise. For distances greater than two centimeters, measuring MMG of voluntary contractions in medium-sized muscles with current OPM technology and conventional magnetic shielding cannot be recommended.

## Introduction

Since 1972, there has been interest in measuring the magnetic field of skeletal muscles^1^. In comparison to the conventional electrophysiological measure of muscle activity, i.e., surface electromyography (sEMG) and needle electromyography (nEMG), which require uncomfortable skin preparation and can be prone to skin breakdown and pain, MMG offers a contactless and non-invasive alternative^2–10^. While both EMG and MMG signals originate from the same biophysical source, i.e., muscle fiber action potentials, and exhibit similar temporal and spectral characteristics^1,11^, MMG might be superior over EMG regarding the decomposition of motor unit units^12^. Decomposition performance depends on the signal-to-noise ratio (SNR)^13^, which depends on the distance between the sensor and the source. Although this relationship has been recognized in experimental MMG studies as a crucial factor to consider^4,5,10,11,14^, it has yet to be studied systematically.

With recent technological advancements, miniaturized optically pumped magnetometers (OPM) have emerged as a new tool to measure MMG. OPMs are quantum sensors utilizing the Zeeman effect and a zero-field resonance. State-of-the-art commercially available zero-field OPMs have sensitivities of up to 10 to 20 ft/$\sqrt{Hz}$ (see also^15–17^) and have been utilized to measure biomagnetic signals from the brain, heart, nerves, and muscles^5,18–24^. Compared to expensive and large superconducting quantum interference devices (SQUIDs), OPMs are a potentially smaller and less expensive alternative that eliminates the need for cryogenic cooling (i.e., -269 °C for liquid helium). The cryogenic cooling for SQUIDs results in sensor-to-body distances of at least a few centimeters, whereas OPM allow a closer sensor placement^5,17,25–27^. This possibility of adaptive sensor placement is especially advantageous for MMG, which is why the effect of sensor-to-source distance is a particularly relevant factor to consider^19,28^.

Moreover, one needs to consider the three-dimensional geometry of muscle fibers and corresponding magnetic fields^29^ in relation to the spatial orientation of sensors. While the axes of OPM can practically not be perfectly aligned with the muscle anatomy for in vivo experiments, in silico studies can overcome these limitations. Thus, we performed a comparative analysis of both in vivo and in silico experiments to characterize the influence of sensor-to-source distance on magnetic muscle signals using OPM-MMG with simultaneous sEMG and an established muscle model^30,31^.

## Materials and methods

A comparative in vivo (OPM-MMG) and in silico study of the effect of sensor-to-source distance was performed.

### Sensor-to-source distance

**Fig. 1:**
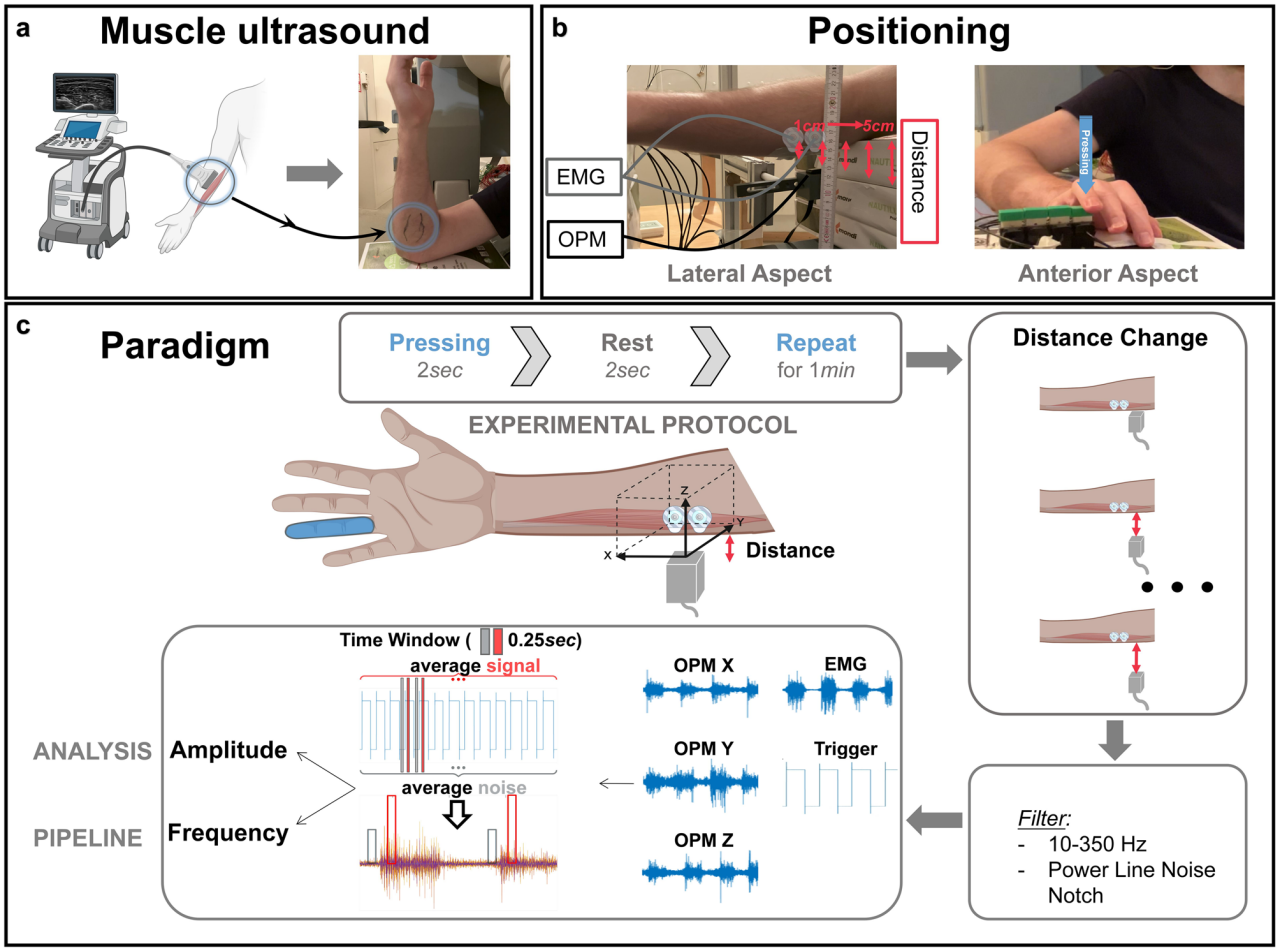
(**a**): After muscle ultrasound, the area of the flexor muscles of the ring finger was marked to position OPM and EMG optimally at the muscle belly. (**b**): The right arm was positioned horizontally between two equally elevated surfaces. The OPM was placed directly below the center of the marked area, and bipolar non-magnetic surface electrodes were positioned directly above the OPM. Each time the subject pressed the button, the trigger channel generated and recorded a trigger signal. (**c**): Three vector components were measured (X, Y, and Z). We identified trigger event peaks to ensure accurate signal and noise time windows aligned with press and rest periods. A noise time window (grey) was defined as the 0.25-s width starting 0.5 s before the peak, while a 0.25-s width signal time window (red) started 0.5 s after the peak. Each signal and noise window was carefully inspected to confirm their precise alignment with press and rest periods. After that, the average root-mean-square(RMS) as a metric of amplitude and the MDF were calculated.

**Fig. 2:**
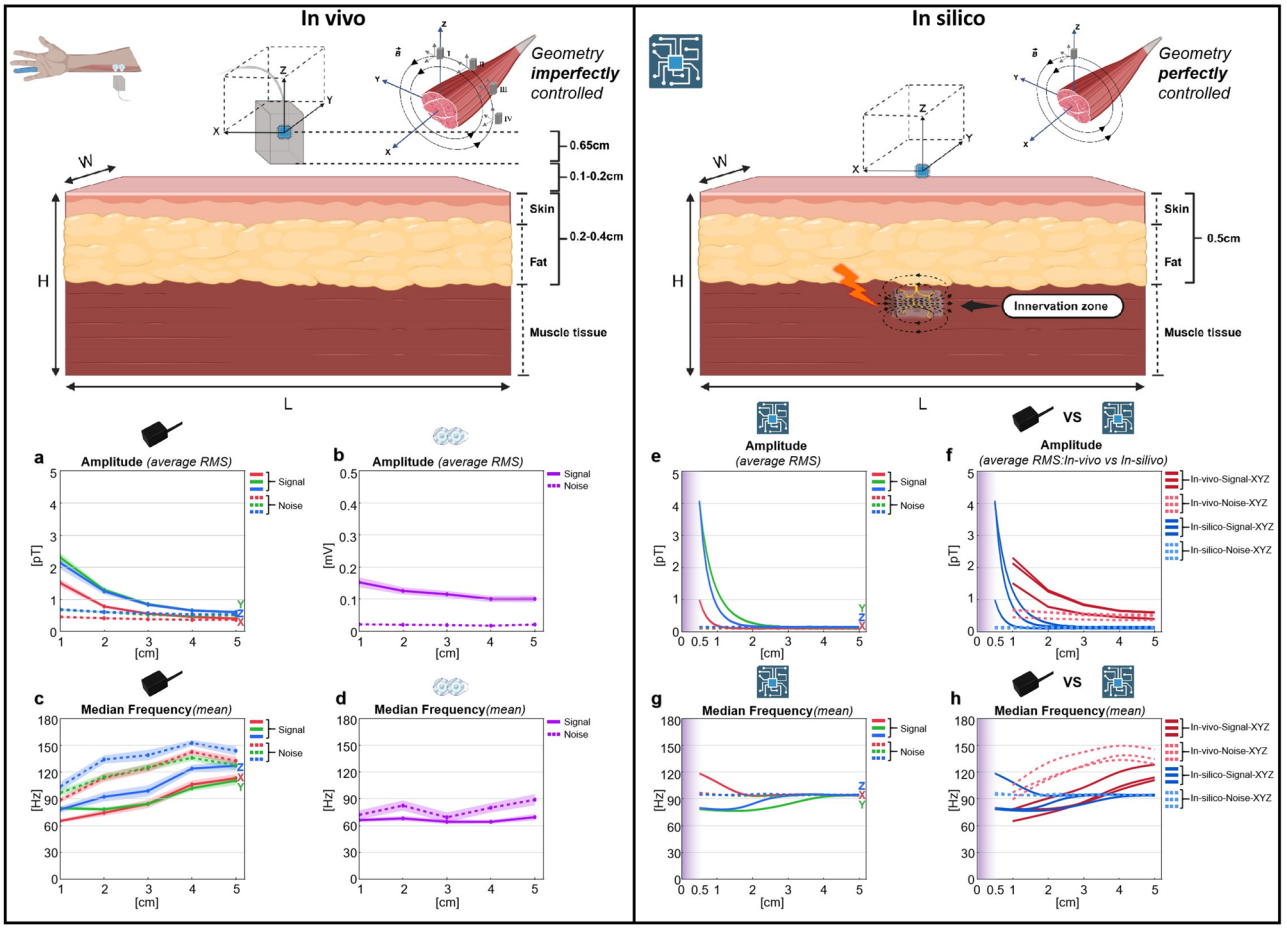
Comparison between the in vivo (left, including standard error) and in silico (right) experiments regarding amplitude and frequency with the corresponding distances based on sensing cell (blue cube). We defined the direction as follows: X aligned with the muscle’s main axis of action, i.e., from one tendon to the other; Y orthogonal to the longitudinal muscle fiber direction, perpendicular to the sagittal plane of the body; Z pointing towards the muscle’s cross-sectional center, i.e., comparable to cross-sectional. (**A**) The average RMS of the three orthogonal components of the magnetic field vector and noise; (**B**) The average RMS of EMG signal and noise; (**C**) The mean MDF of the three orthogonal components of the magnetic field vector and noise; (**D**) The mean MDF of EMG signal and noise. Note that the EMG electrodes have consistently remained attached to the skin, i.e., the testing distance of EMG did not change. This representation is included only to demonstrate that the testing distance remains nearly constant. In silico experiments: (**E**) The average RMS and noise; (**F**) Comparison of the average RMS between in vivo and in silico and – given a skin and subcutaneous fat thickness of 0.5 cm-the highest RMS and thereby SNR can be expected below 1 cm; (**G**)The mean MDF and noise; (**H**) Comparison of the mean MDF between in vivo and in silico. Note that the purple shaded area marks mean it is impossible to measure MMG signals at these distances in the current experiment and require new sensors, i.e., the distance between skin and muscle. Additionally, for a–f, the in vivo and in silico results are presented without any fitting. We applied the spline smoothing function (see also methods) in JMP to generate the best-fit curve that captures the underlying trends in the data for a better comparison, as shown in g and h.

The sensor-to-source distance *r* refers to the distance between the skin and the sensing point, as shown in Fig. 1 (in vivo) and Fig. 2 (in silico). Here, the used OPM (QZFMgen-3, QuSpin Inc., Louisville, CO, United States) with sensing cells (vapor cell) 0.65 cm distant from the outer shell of the OPM, which adds up to a minimal 0.1—0.2 cm OPM-shell-to-skin distance to ensure that the skin and the OPM do not contact during muscle contraction. Adding an experimental variance of 5% when placing the OPM closer or more distant to the skin and the skin-fat thickness of about 0.2—0.4 cm – the minimum distance between the sensor (OPM vapor cell) and source (muscle tissue) will be 1 cm for in vivo experiments (Fig. 2). In silico and in vivo experiments were compared at the same sensor-to-source distance. Moreover, in silico experiments also allowed predictions below the nearest possible in vivo sensor-to-source distance.

Although EMG has been consistently used as the gold standard for measuring neuromuscular signals, OPM-MMG and EMG can measure similar muscle signals during muscle activity, as shown by several studies^4,6,32^. In our study, we focus on how changes in the sensor-to-source distance affect OPM-MMG for measuring neuromuscular signals. In contrast, for EMG, the distance between the electrode and the signal source is constant since the EMG electrodes necessitate skin contact. This clearly contrasts OPM-MMG, where the distance between the sensor and the signal source can vary. As a result, EMG can serve as a “control modality” as the sensor-to-source distance does not vary.

### In silico experiment

We conducted computer simulations using an established continuum multiscale approach previously published by Klotz et al.^30,31^. This model simulates the muscle fiber action potentials and the corresponding EMG^31^. With the solution of the electrophysiological muscle model at hand, one can compute the muscle-induced magnetic field using the Biot-Savart law^33,34^. Here, we used a cube-shaped layered tissue model consisting of a muscle with edge length L = 6 cm, W = 1.5 cm, and H = 2 cm. The cubic model contains a single motor unit, which provides a simplified view of neuromuscular activity, in contrast to the in vivo MMG studies involving multiple motor units. Further, a layer of subcutaneous fat with a thickness of 0.2 cm is added on top of the muscle. The virtual motor unit response was simulated by considering a motor unit territory at a depth of 0.5 cm and centered in the lateral direction of the muscle. The muscle-induced magnetic field was computed for a set of virtual magnetometers in a line orthogonal to the body surface between the innervation zone and the myotendinous junction (Fig. 2). Further, the sensor-to-source distance was between 0.5 and 5 cm, with increments of 0.1 cm. We considered 100 repetitions of the compound muscle response to study the statistical effect of noise. The interval between the stimuli was 100 ms (with a random jitter of + /- 10%). We used additive, filtered Gaussian noise to approximate the experimental situation in the computer simulations. In detail, the OPM was modelled as a first-order low-pass filter with a cut-off frequency of 130 Hz. Moreover, as for the experimental signals, we applied a 4th-order bandpass filter between 10 and 350 Hz. At a virtual sensor-to-body distance of 0.5 cm, the normalized noise amplitude was assumed to be equivalent to the average noise level of the in vivo MMG. We computed the simulated signal’s average root-mean-square (RMS) and median frequency (MDF) for the cases with and without additive noise. Afterwards, the signal-to-noise ratio was calculated by dividing the difference between the average RMS of the signal and the average RMS of the noise by the average RMS of the noise.

### In vivo experiment

Three healthy subjects participated in the study (3 males; mean age: 36.7 ± 4.0 SD years; and mean body-mass-index: 22.7 ± 0.4 SD kg/m^2^). The experiment was conducted at the MEG Center of the University of Tübingen (Germany) in December 2023, inside a magnetically shielded room (Ak3b, VAC Vacuumschmelze, Hanau, Germany), according to the standards of the World Medical Association. All participants were authors of this publication and gave informed consent to publish their data. The study was conducted in accordance with the Declaration of Helsinki and approved by the ethics committee of the University of Tübingen.

Before the measurement, ultrasound imaging (Mindray TE7, 14 MHz-linear probe) of the flexor digitorum superficialis was performed for each subject by a board-certified ultrasound user (JM) to determine the longitudinal axis of the finger flexor muscles of the ring finger, i.e., the signal source (Fig. 1a). All participants were comfortably seated, with their right arm positioned horizontally between two equally elevated surfaces (Fig. 1b). The OPM and bipolar EMG electrodes were placed according to the previously measured muscle ultrasound (Fig. 1b). During the experiment, participants were instructed to press a button downward self-paced using their ring finger and maintain the press for 2 s at an individual, stable, and convenient force level, followed by a brief 2 s rest. Force was not recorded. This cycle was repeated until a 60 s recording was completed. The OPM was initially placed approximately 0.1—0.2 cm away from the identified point of the skin, i.e., the outer-shell-to-skin distance was 0.65 cm, which implies a sensor-to-source distance of 1 cm. After each 60-s trial, the OPM was systematically moved further from the skin in 1 cm steps, resulting in five different sensor-to-skin distances.

A custom-built plastic frame attached to a non-magnetic aluminum structure held the OPM. The OPM simultaneously recorded three orthogonal components of the magnetic field vector, denoted as X, Y, and Z. We aligned the OPM as follows: The X-direction was parallel to the longitudinal muscle fiber direction; the Y-direction was orthogonal to the longitudinal muscle fiber direction. The Z-direction points towards muscle fiber, i.e., comparable to cross-sectional (Fig. 1c). Additionally, bipolar non-magnetic surface electrodes (Conmed, Cleartrace2 MR-ECG-electrodes) were positioned directly below the OPM, with a 1 cm distance between them (Fig. 1b). The ground electrode was placed on the back of the neck, on the 7th cervical vertebra. To our knowledge, no non-magnetic disposable EMG electrodes are commercially available. Therefore, we attached MR-ECG electrodes by twisting a paramagnetic cable wire around the electrode button^9^. This setup allowed simultaneous recordings of both EMG and OPM-MMG.

The data acquisition system of the Tübingen MEG-system (CTF Omega 275, Coquitlam, BC, Canada) was used to simultaneously record the OPM-MMG, sEMG, and the button press trigger signal. The Electroencephalogram (EEG) channels included in the system were used to acquire the sEMG data. Both the OPM-MMG and sEMG data were sampled at a rate of 2,343.8 Hz. The OPM utilized in this study had a magnetic field sensitivity of 15 fT/$\sqrt{Hz}$ and a 3 to 135 Hz bandwidth. It could operate below 200 nT and had a dynamic range of a few nT. The OPM was equipped with internal compensation coils^35^ to accommodate any magnetic background field. These coils could effectively cancel magnetic background fields up to 200 nT within the sensing hot rubidium vapor cell, which has 3 × 3 × 3 mm^3^ dimensions. For all measurements, an output gain factor of 3 was applied in the user interface of the sensors, which corresponds to a conversion factor for the analog output of the OPMs to a magnetic flux of 2.7 V/nT.

### Preprocessing and data analysis

Data preprocessing was performed using Matlab R2022b (The MathWorks, Natick, MA, USA), the FieldTrip toolbox^36^ and custom scripts. After demeaning, sEMG and OPM-MMG datasets were filtered with 10 Hz high-pass and 350 Hz low-pass 4th-order Butterworth filters^37^. The spectrum was normalized to identify abnormal peaks (such as 50 Hz or 16.67 Hz (trains) power-line noise) based on the peak frequency and remove them automatically by a Matlab custom script. We relied on the recorded trigger channel to partition the signal.

During the experiment, the duration of press and rest periods for each subject varied slightly from the intended 2 s but consistently exceeded 1 s. We identified trigger event peaks to ensure accurate signal and noise time windows aligned with press and rest periods, respectively. A noise time window (Fig. 1c, grey) was defined as the 0.25 s interval starting 0.5 s before the peak, while a 0.25 s signal time window (Fig. 1c, red) started 0.5 s after the peak. Each signal and noise window was carefully inspected to confirm their precise alignment with press and rest periods. Outliers were defined as windows with root-mean-square (RMS) and median frequency (MDF) values exceeding three times the median absolute deviation from the median. MDF and RMS were calculated for each window as parameters of interest. We averaged MDF, RMS, and noise values across the three participants to obtain mean MDF, average RMS, and average noise. The same data processing methods were applied to the sEMG data. Meanwhile, a consistent signal-to-noise ratio calculation approach was utilized in the in vivo experiment.

Median frequency is an important spectral parameter that helps describe the muscle activity state^38,39^. Compared to mean frequency, median frequency is more effective in avoiding interference from noise in spectral analysis. Mean frequency (MF) is the weighted average of all frequencies in the spectrum and is prone to the influence of extreme values (such as noise or outliers). Particularly in MMG signals, the impact of noise and extreme values can cause shifts in the mean frequency, making the analysis less accurate. In contrast, median frequency is better at avoiding the influence of these extreme values, providing more stable and reliable results.

### Statistics

The SPSS Statistics version 25 (IBM Corporation, Armonk, NY, USA) was used for statistical testing. The signals from multiple time windows were averaged at varying sensor-to-source distances for each subject. Average RMS and mean MDF in the X, Y and Z directions were then compared across different sensor-to-source distances using the “multicompare” function. ANOVA with Tukey’s method was used to compare the effect of these different sensor-to-source distances. All visualisations were performed using GraphPad PRISM 9.5 (GraphPad Software Inc.), and p < 0.05 was considered significant. The Shapiro–Wilk test was performed to test if the small sample data followed a normal distribution, and p > 0.05 indicated normality.

We used JMP (version 16, SAS Institute Inc., Cary, NC, USA) to visualise our data and present the standard errors in the figure, as well as the spline smoothing function to fit the data. Spline smoothing employs a piecewise polynomial approach, dividing the data into multiple intervals (referred to as “knots”) and fitting a low-order polynomial (commonly cubic polynomials) within each interval. Continuity constraints are imposed on the function’s value and first and second derivatives to ensure a smooth transition between segments. JMP automatically determines the knot placement and optimal smoothing parameter (lambda) to generate the best-fit curve that captures the underlying trends in the data.

## Results

The in vivo and in silico results are shown in Figs. 1–3 and Supplemental Figs. 1–5 and can be found in Supplemental Tables 1 and 2.

**Fig. 3:**
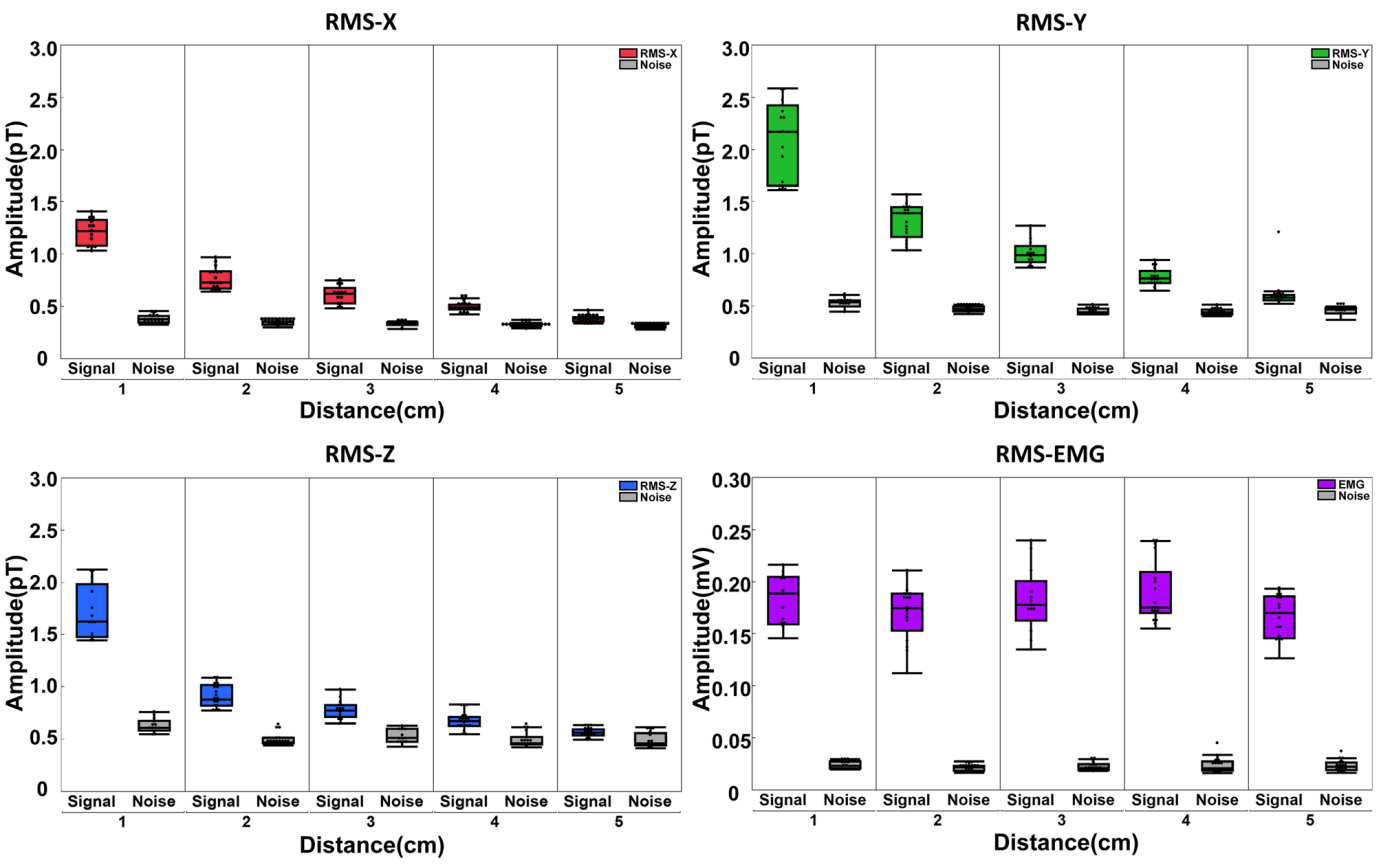
Illustration of the distribution of the RMS during button press (signal) and rest (noise) of a representative subject No. 1 at different sensor-to-source distances both in OPM-MMG (RMS X, Y, and Z) as well as EMG.

### In vivo experiment

The average signal RMS at 0.5–1 pT noise level from 10 to 350 Hz decreased in X, Y, and Z directions as the sensor-to-source distance increased (Fig. 2a). Shapiro–Wilk tests did not reject the normality of RMS across subjects in the X, Y, and Z directions (P_x_ = 0.06, P_y_ = 0.12, P_z_ = 0.12). ANOVA on average RMS across all subjects revealed a significant effect of distance for all directions (F(4,30) = 10.55, p < 0.05, Supplemental Fig. 2a). At the same time, there was no statistically significant difference in average MDF across different sensor-to-source distances (Supplemental Fig. 2b). The mean RMS at 1 cm (100%) was 1.51, 2.31 and 2.13 pT and reduced to 0.78, 1.29 and 1.25 pT at 2 cm (51.66%, 55.84% and 58.69%) for the X, Y and Z direction, respectively. Thus, when the sensor-to-source distance increased from one to two centimeters, the mean RMS decreased by about 45% across all directions. Beyond 2 cm distance, the RMS reduction became less significant (Supplemental Table 1).

In contrast to signal RMS, the noise RMS remained nearly unchanged at 0.5–1 pT from 10 to 350 Hz for different distances. The magnetic muscle signal (averageRMS) was highest in Y (orthogonal to the muscle fibers and tangential to the body surface) and lowest in the X direction (parallelly aligned with the longitudinal muscle fiber direction). Once the distance exceeded 2 cm, the average signal RMS was close to the noise level (Fig. 2a). This was evident for the average RMS across all subjects (Fig. 2a) and for individual subjects (Fig. 3). Conversely, the signal median frequency (MDF) of OPM demonstrated an increasing trend in all directions and ultimately approached the MDF of the noise.

The sensor-to-source distance remained constant for EMG as the electrodes were permanently attached to the skin. Nevertheless, the average RMS in EMG showed a slight decrease with distance, possibly due to individuals applying lower pressure, while the mean MDF remained nearly unchanged. The average RMS and mean MDF in EMG showed no significant statistical differences regarding distance changes.

### In silico experiment

The simulations allow the decomposition of the total MMG signals into (1) additive noise (also referred to as “pure noise”) and (2) pure signal (see Supplemental Fig. 3). For the total simulated signal (i.e., including noise), we observed a decreasing trend of the average RMS in all three directions (X, Y and Z) as the sensor-to-source distance increased. Here, the average RMS was the highest in the Y-direction and lowest in the X-direction, consistent with the in vivo experiments. Increasing the sensor-to-source, the average RMS approached the noise level (Fig. 2e). At a sensor-to-source distance of 2 cm, the SNR is 12.09%, 15.90%, and 4.62% of the value at 1 cm in the X, Y, and Z directions, respectively.

The mean MDF demonstrated an increasing trend in the Y-direction and Z-direction and a decreasing trend in the X-direction as the sensor-to-source distance increased, eventually approaching the noise MDF (Fig. 2g). Furthermore, the mean MDF demonstrated by the in silico experiments was higher than that observed in the in vivo experiments when the sensor-to-source distance was below 2 cm. At the same time, we observed that the MDF of the “pure signal” (signal without noise) showed a decreasing trend in the X, Y and Z-directions, and this trend was strongest in the X-direction, with only a slight decrease in the Y and Z-directions (Supplemental Fig. 3).

### Signal-to-noise ratio

We calculated the SNR of OPM-MMG datasets in both in vivo and in silico experiments (Supplemental Table 1, Supplemental Fig. 4) and the SNR of EMG datasets in the in vivo experiment (Supplemental Table 2). Whereas the SNR between the in vivo and in silico experiments for different sensor-to-source distances were similar, the in silico experiments revealed that the highest SNR could be expected for sensor-to-source distances below 1 cm (Supplemental Fig. 4). The SNR gradually approached zero for sensor-to-skin distances above 2 cm (Supplemental Table 1).

## Discussion

To the best of our knowledge, this study is the first to characterize the effect of sensor-to-source distance on the MMG signal utilizing a combined in vivo and in silico approach. The low number of participants was due to the proof-of-principle concept of this study.

Our findings show that, when analyzing voluntary MMG signals from muscles that are similar in size and anatomy to FDS at a noise level of 0.5–1 pT for 10–350 Hz, the MMG signal tends to reach the ambient magnetic noise level when the sensor-to-source distance exceeds 2 cm (Fig. 2 and 3, Supplemental Table 1 and 2). This suggests that the magnetic activity of medium-sized muscles, such as FDS, cannot be reliably recorded beyond a two-centimeter distance at given noise levels at the considered contraction intensity. In addition to the limitations of OPM itself, the shielding environment also restricts the detection of muscle signals at greater distances as it influences the overall magnetic noise floor; here, we performed our experiments in a magnetically shielded chamber with a noise floor of 10 ft/$\sqrt{Hz}$. Our study took the first step toward investigating the relationship between sensor and source in MMG.

Given the decrease (also see the previous studies^5,14,40^) in signal amplitude over distance, one can expect the highest SNR for a distance below 1 cm, i.e., for the actual minimal sensor-to-source distance in vivo (Supplemental Fig. 4). The in vivo SNR at 1 cm (100%) was 2.33, 2.44 and 2.10 and reduced to 0.89, 1.16 and 1.06 at 2 cm (38.20%, 47.54% and 50.48%) for the X, Y and Z direction, respectively. Especially for such small distances, every millimeter of lower distance results in a significant increase in the SNR (Supplemental Table 1). If it was possible to position the actual sensing cell closer to the source, one could also compromise on a lower sensor sensitivity. This is relevant for future technological developments in the field of magnetometers, as exemplified by Gherardini et al.^41^, which demonstrated wearable magnetic technology for real-time monitoring of muscle activity. Minimizing the sensor-to-source distance in in vivo experiments is anticipated to result in higher SNR comparable to those achieved with sEMG (Supplemental Fig. 4).

Meanwhile, we performed a normalized comparison of RMS between the in vivo and in silico experiments at the same sensor-to-source distances. When the sensor-to-source distance increased from 1 cm (100%) to 2 cm, the normalized RMS in the in vivo experiments decreased by 66.07%, 59.59%, and 57.47% in the X, Y, and Z directions, respectively. In contrast, the normalized RMS in the in silico experiments decreased by 95.22%, 87.94%, and 96.21% in the X, Y, and Z directions, respectively (Supplemental Fig. 5). A plausible explanation is that we rely on an empirical estimate of signal amplitude experimentally since only the combined signal and noise can be measured.

A remarkable difference between the in vivo and in silico results is that the in silico model can operate in perfect conditions and, e.g., can quantify the MMG signal at 0.5 cm distance, which is not feasible in vivo with the OPM technology used in our study. However, our in silico model lies in its inability to perfectly reflect the complexities of real-world conditions, such as underrepresenting the anatomical complexity of multiple motor units and their contribution to the measured signal. Furthermore, the model and our measurements do not consider several factors that may affect the MMG signal, thus pointing to fruitful questions for future studies.

### Limitations

The limitations of our experimental setup remain that should be addressed systematically in future studies: Firstly, the measurements were conducted on only three healthy subjects. While the findings provide proof of principle, explicitly highlighting the significance of the sensor-to-skin distance in the overall experiment and simulation experiments supported the results, validating the findings and estimating group differences through larger sample sizes is necessary. Secondly, the sensor-to-source distances were adjusted in centimeter-scale increments in our in vivo experiments, in contrast to the millimeter-scale increments used in silico experiments. Future studies could explore the use of finer divisions for sensor-to-source distances.

While the computer model is particularly useful for understanding the basic properties of magnetic muscle signals comprehensively (here, the signal decay and modulation of the frequency content), it has several limitations. Although previous studies (Klotz, 2022^30^) have validated its use, it is important to recognize that the utilized cube-shaped tissue geometry misses the effect of the (often complex) fibre architecture in real muscles. The effect of the tissue anatomy needs to be addressed in the future, e.g., using the finite element method. Moreover, in the presented simulations, we focused on the MMG signal generated by a single motor unit. However, in voluntary contractions, a whole pool of motor units is active. Finally, it is noted that the model parameters are adopted from the literature and do not represent individual subjects.

### Geometrical orientation between the sensor and the source

Given the current knowledge that most of the current during muscle activity flows along the muscle fibers’ longitudinal direction, the three-dimensional angle (i.e., the angle of muscle fibers according to the longitudinal axis of the respective muscle) becomes relevant when measuring MMG. This circumstance was already noted^14^ and shown^3,32^ before. In line with these studies, both our in vivo and in silico experiments show the largest signal amplitude (here, the average RMS) in the Y-direction, i.e., orthogonal to the muscle fibers and the lowest signal amplitude in the X-direction, i.e., aligned with the muscle fibers. Furthermore, we observed that the average RMS in the X-direction was significantly lower in the in silico experiment compared to in vivo when the sensor-to-source distance was 1 cm. This difference could be attributed to the perfectly controlled geometry of the in silico model, leading to a lower average RMS that parallelly aligned with the longitudinal muscle fiber direction. This can also be explained by an imperfectly controlled OPM positioning in relationship to the angle of the muscle fiber of FDS (also illustrated in Fig. 2), as a permanent tracking of the geometrical orientation between the sensor (OPM) and the source was impossible. Furthermore, it is highly improbable that the OPM axis is perfectly aligned with the muscle geometry. The muscle fibers may still exhibit slight curvature even if local alignment is achieved.

In our in silico experiments, we observed that the mean MDF in the X-direction did not increase with increasing sensor-to-source distance, unlike the in vivo experiments where it increased. A plausible hypothesis is that, in all cases, with increasing distance, the mixed signal approaches the median frequency of the noise. For the Y- and Z-direction, the median frequency at the lowest distance is lower than for the noise. Hence, the MDF increases with increasing distance. In contrast, for the X-direction, the median frequency at the lowest distance is higher than for the noise. To match the noise value at higher distances, it decreases until it meets the median frequency of the noise. This could also explain the differences in median frequency between in vivo and in silico. Additionally, it might be possible that the triaxial OPM’s measured direction (X, Y or Z) is influenced by a different sensitivity per axis^29,42^. Moreover, the MDF of the “pure signal” demonstrated a decreasing trend in the X, Y, and Z directions, which enabled us to predict variations in signal frequency based on the sensor-to-source distance, which was impossible in the in vico experiment due to the increasing influence of noise. This could also explain that increasing the MDF value of the MMG signal in vivo is due to the increased interference of the noise as the sensor-to-source distance increases.

### Continuous distance measurements

In contrast to MEG studies, where the signal source does not inherently move, the source changes its volume in MMG due to mechanical deformation during muscle contraction. Consequently, the sensor-to-source distance changes depending on the muscle studied. As muscle deformation can also change the muscle angle^3,32^, continuous tracking of muscle volume, distance, and angle should be incorporated in future studies.

### Influence of muscle force

Also, force influences the MMG signal. The more force a muscle generates, the stronger the MMG signal becomes as more motor units are recruited. Moreover, increasing muscle activity increases the (destructive) interference level, yielding a complex force-amplitude curve. Although this has yet to be studied in MMG, one can assume that MMG does not behave differently from EMG, where the connection between force and signal strength is already far better understood^43–45^.

Although the force intensity was not monitored in our in vivo experiments, we calculated the average RMS of the EMG datasets and observed negligible changes in the RMS with the sensor-to-source distance change. Given the almost unchanged signal EMG-RMS (Fig. 2b), we can assume that all subjects maintained their individual force level during the experiments. If not averaging as we did in our in vivo experiment, the force applied should be recorded simultaneously. However, they probably had notably different force levels during button press, as subject 1 had the highest EMG amplitude throughout all experiments (Supplemental Fig. 1). Thus, we suggest monitoring force and systematically different force levels at the best possible distances, using force feedback for the performing subject.

### Motor unit decomposition & invasive EMG studies

Motor unit action potentials are the atomic components of the MMG signal. The latter is, in fact, nothing else than the result of the asynchronous activation of several motor units of different sizes. Decomposing motor units means understanding the real corticospinal drive to the muscle and can be helpful in diagnostics, monitoring, and prosthetics control. Consequently, if MMG is used for such purposes, it would be reasonable to investigate to what extent motor unit decomposition can be performed at different sensor-to-source distances. Our study had only one OPM available, effectively preventing motor unit decomposition. However, it should be noted that in silico studies have already shown the potential superiority of MMG compared to EMG for motor unit decomposition^46^.

The “ground truth” and the gold standard for studying muscle activity is invasive EMG, which allows the accurate detection of single motor units and, hence, the essential signal sources of voluntary MMG. Consequently, a simultaneous recording of the gold standard of muscle activity would be preferable when studying sensor-to-source distance in MMG. Ideally, such measurements should also include a high-density invasive EMG, as shown by Muceli^47^, which can record up to 50 motor units simultaneously and thereby accurately localise different motor units during muscle activity. However, such invasive EMG electrodes are currently magnetic and thus cannot be used for simultaneous invasive EMG and MMG.

### Improvement of signal-to-noise ratio

Currently, there is still great potential for future technological advancements to improve the signal-to-noise ratio (SNR) of MMG recordings. Most obviously, (1) enhancing the sensor’s sensitivity. However, the optimal balance between sensitivity and bandwidth remains unclear in OPM-MMG, as increasing OPM sensitivity typically reduces bandwidth and vice versa. (2) If future technological advancements decrease the distance between the sensing vapor cell and the outer shell of the OPM, this would shorten the sensor-to-source distance, resulting in a notable improvement in the SNR. (3) Optimising the shielding environment can further minimize interference caused by noise. One advantage of MMG over EMG is its ability to use miniaturized shielding to effectively block all forms of environmental noise, including electrical interference from electrical stimulation, allowing artifact-free recordings^48^.

The SNR is crucial for extracting motor unit action potentials from EMG signals. Zaheer et al.'s^13^ study indicates that a minimum SNR of 3 is required for reliable motor unit yield in surface EMG decomposition. In our study, the optimal in vivo SNR of the OPM sensor was 2.44 (direction-Y), which is already close to this value. Moreover, recent studies have demonstrated the importance of SNR optimization in improving gesture recognition performance across different sensing technologies in practical applications^49,50^. Meanwhile, Greco et al.^4^ have already shown the feasibility of OPM sensors for contactless finger muscle activity recognition. In conclusion, improving the SNR in MMG-based neuromuscular signal measurements will be pivotal in future technological applications.

## Conclusion

For the sensor-to-source distance above two centimeters, measuring voluntary MMG of medium-sized muscles such as the FDS will be almost impossible with current OPM and shielding technology.

## Supplementary Information

Supplementary Information 1.

Supplementary Information 2.

Supplementary Information 3.

Supplementary Information 4.

Supplementary Information 5.

Supplementary Information 6.

Supplementary Information 7.

Supplementary Information 8.

## Data Availability

The original data can be made accessible upon reasonable request directed to the corresponding author.

## Abbreviations

MMG: Magnetomyography
OPM: Optically pumped magnetometer
OPM-MMG: Optically pumped magnetometer-based magnetomyography
EMG: Electromyography
FDS: Flexor digitorum superficialis
RMS: Root mean square
MEG: Magnetoencephalography
SQUID: Superconducting quantum interference device
sEMG: Surface electromyography
nEMG: Needle electromyography
MDF: Median frequency
SNR: Signal-to-noise ratio
