# Supplementary figures and images for: The effect of sensor-to-source distance on magnetic neuromuscular signals
Source: Sci Rep. 2025 Jun 20;15:20225. doi: 10.1038/s41598-025-06545-1 (PMC12181354; doi:10.1038/s41598-025-06545-1)

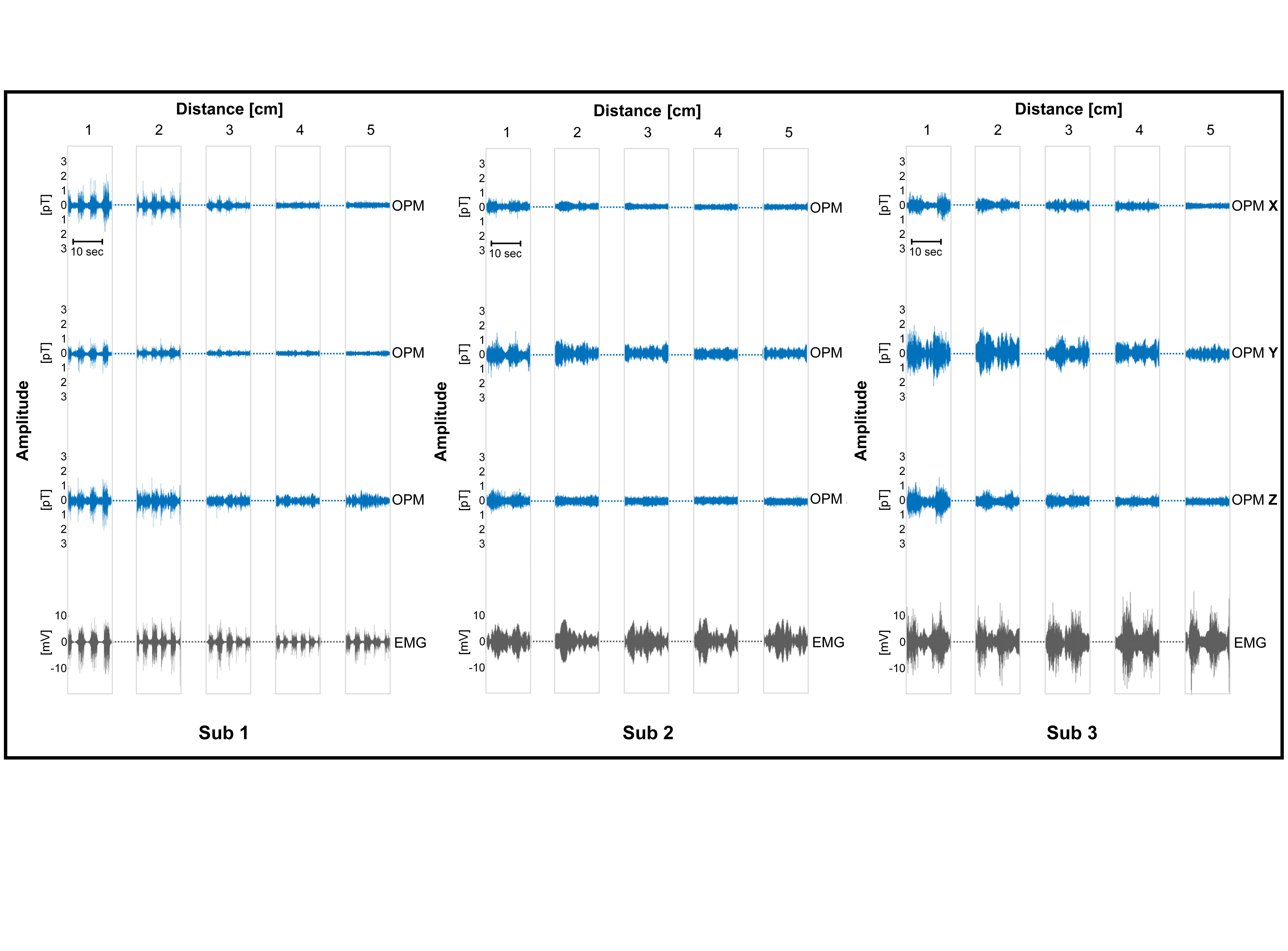

Supplement: Supplementary file 1 — Supplementary Information 1. [file 41598_2025_6545_MOESM1_ESM.jpg]

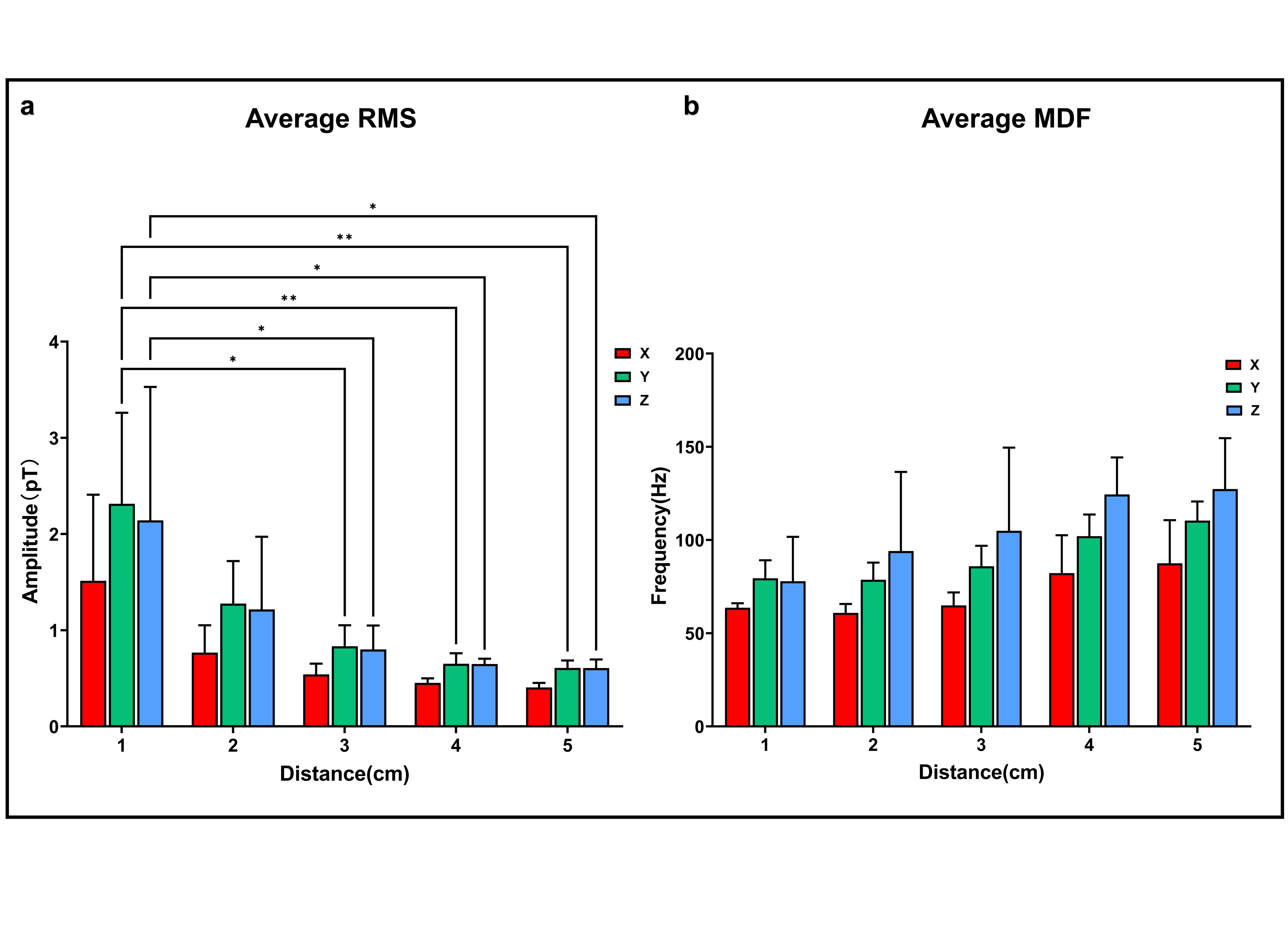

Supplement: Supplementary file 2 — Supplementary Information 2. [file 41598_2025_6545_MOESM2_ESM.jpg]

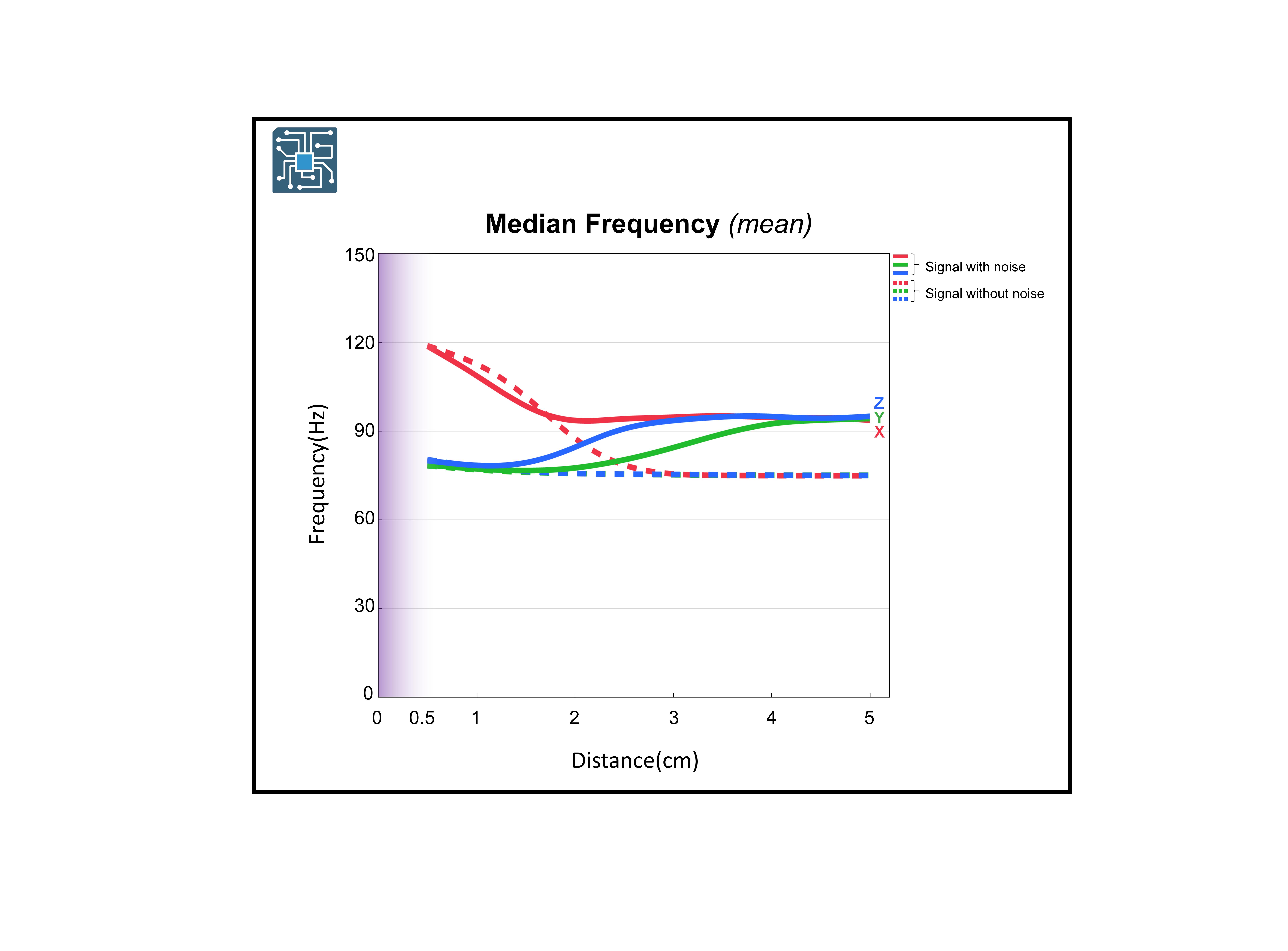

Supplement: Supplementary file 3 — Supplementary Information 3. [file 41598_2025_6545_MOESM3_ESM.jpg]

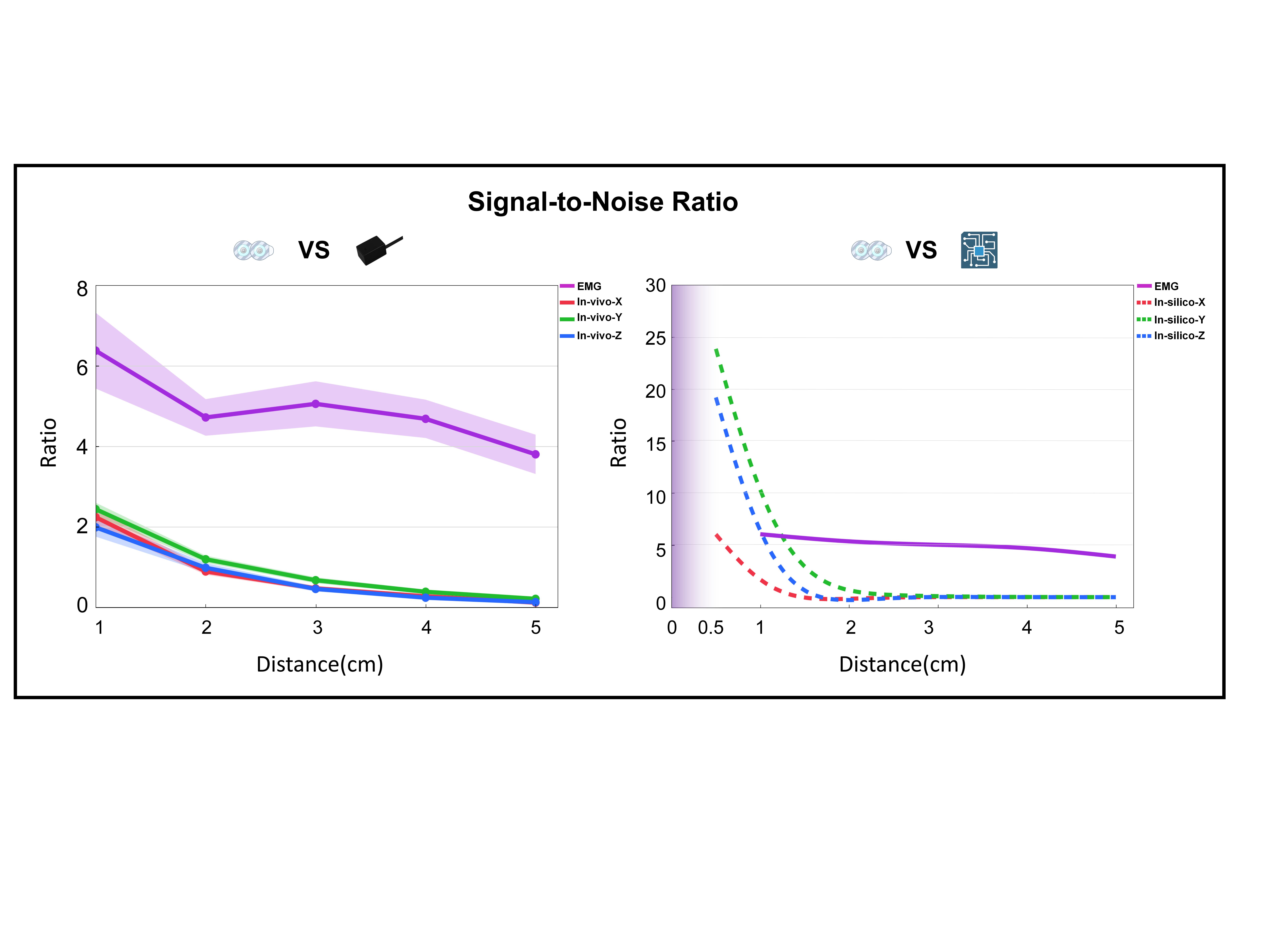

Supplement: Supplementary file 4 — Supplementary Information 4. [file 41598_2025_6545_MOESM4_ESM.jpg]

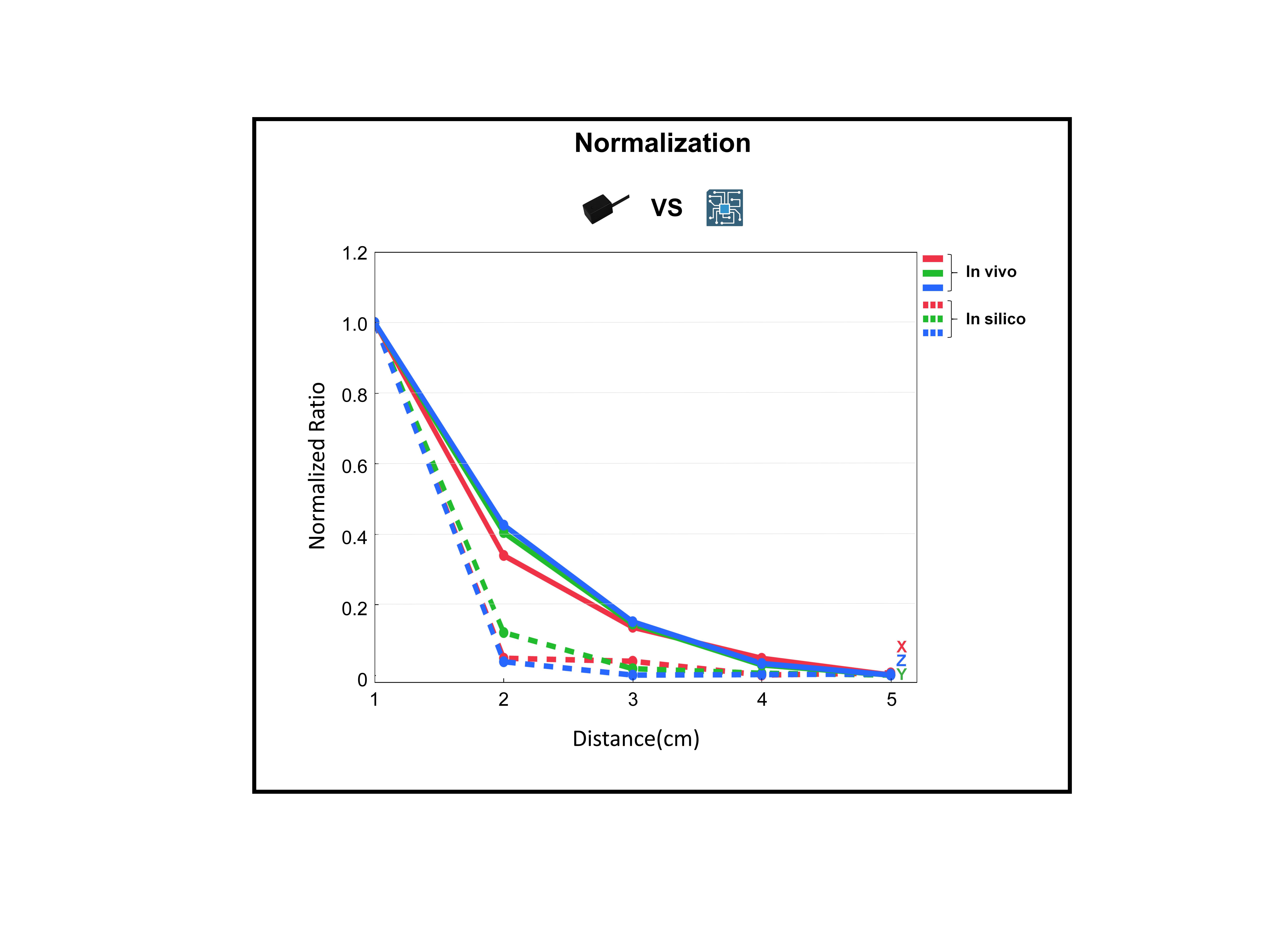

Supplement: Supplementary file 5 — Supplementary Information 5. [file 41598_2025_6545_MOESM5_ESM.jpg]
