# Supplementary material for: The effect of sensor-to-source distance on magnetic neuromuscular signals
Source: Sci Rep. 2025 Jun 20;15:20225. doi: 10.1038/s41598-025-06545-1 (PMC12181354; doi:10.1038/s41598-025-06545-1)
Supplement: Supplementary file 6 — Supplementary Information 6. [file 41598_2025_6545_MOESM6_ESM.docx]

***Supplemental Figure 1*:** OPM-MMG and EMG datasets of the three subjects within the 10-second window in all sensor-to-skin distances.

***Supplemental Figure 2*:** Comparison of the average signal at different sensor-to-source distances. (A) Average RMS of X, Y, and Z at different sensor-to-source distances. (B) Average MDF of X, Y and Z at different sensor-to-source distances. Error bars indicate the standard error of the mean across participants (*p<0.05, **p<0.01).

***Supplemental Figure 3*:** Comparison of the mean MDF of the signal with noise and the "pure signal".

***Supplemental Figure 4*:** Comparison of signal-to-noise ratio at different sensor-to-source distances.

***Supplemental Figure 5*:** Comparison of the normalized RMS between in vivo and in silico.

***Supplemental Table 1*:** Signal-to-noise ratio of the average RMS of in-vivo and in-silico experiments at various sensor-to-skin distances of OPM.

***Supplemental Table 2*:** Signal-to-noise ratio values for the average RMS and the MDF performance of EMG. Note that the EMG electrodes have consistently remained attached to the skin, i.e., the testing distance of EMG did not change.
