# Supplementary material for: The effect of sensor-to-source distance on magnetic neuromuscular signals
Source: Sci Rep. 2025 Jun 20;15:20225. doi: 10.1038/s41598-025-06545-1 (PMC12181354; doi:10.1038/s41598-025-06545-1)
Supplement: Supplementary file 7 — Supplementary Information 7. [file 41598_2025_6545_MOESM7_ESM.docx]

| Sensor-to-Source  Distance | Direction | in-vivo | | | in-silico | | |
| --- | --- | --- | --- | --- | --- | --- | --- |
|  |  | RMS(pT) | Noise(pT) | SNR(-) | RMS(pT) | Noise(pT) | SNR(-) |
| 0.5cm | X | - | - | - | 0.9981 | 0.1031 | 8.6809 |
|  | Y | - | - | - | 4.0253 | 0.1315 | 29.6106 |
|  | Z | - | - | - | 4.0872 | 0.1464 | 26.9180 |
| 1cm | X | 1.5115 | 0.4530 | 2.3366 | 0.2539 | 0.1055 | 1.4066 |
|  | Y | 2.3051 | 0.6696 | 2.4425 | 1.5943 | 0.1328 | 11.0053 |
|  | Z | 2.1346 | 0.6878 | 2.1035 | 1.0499 | 0.1537 | 5.8308 |
| 2cm | X | 0.7774 | 0.4119 | 0.8874 | 0.1183 | 0.1011 | 0.1701 |
|  | Y | 1.2914 | 0.5974 | 1.1617 | 0.3704 | 0.1347 | 1.7498 |
|  | Z | 1.2520 | 0.6073 | 1.0616 | 0.1868 | 0.1471 | 0.2699 |
| 3cm | X | 0.5511 | 0.3807 | 0.4476 | 0.1226 | 0.1015 | 0.2079 |
|  | Y | 0.8506 | 0.5180 | 0.6422 | 0.2669 | 0.1316 | 1.0281 |
|  | Z | 0.8311 | 0.5688 | 0.4612 | 0.1585 | 0.1491 | 0.0630 |
| 4cm | X | 0.4532 | 0.3615 | 0.2537 | 0.1173 | 0.1021 | 0.1489 |
|  | Y | 0.6526 | 0.4829 | 0.3514 | 0.2484 | 0.1311 | 0.8947 |
|  | Z | 0.6492 | 0.5317 | 0.2210 | 0.1593 | 0.1491 | 0.0684 |
| 5cm | X | 0.4004 | 0.3623 | 0.1051 | 0.1185 | 0.1021 | 0.1606 |
|  | Y | 0.6039 | 0.5005 | 0.2065 | 0.2499 | 0.1343 | 0.8608 |
|  | Z | 0.5987 | 0.5355 | 0.1180 | 0.1584 | 0.1473 | 0.0754 |

***Supplemental Table 1*:** Signal-to-noise ratio of the average RMS of in-vivo and in-silico experiments at various sensor-to-skin distances of OPM.
