# Supplementary material for: The effect of sensor-to-source distance on magnetic neuromuscular signals
Source: Sci Rep. 2025 Jun 20;15:20225. doi: 10.1038/s41598-025-06545-1 (PMC12181354; doi:10.1038/s41598-025-06545-1)
Supplement: Supplementary file 8 — Supplementary Information 8. [file 41598_2025_6545_MOESM8_ESM.docx]

| EMG in-vivo | | | | | |
| --- | --- | --- | --- | --- | --- |
| Sensor-to-Source  Distance | **RMS(mV)** | **Noise-A(mV)** | **SNR(-)** | **MDF(Hz)** | **Noise-f(Hz)** |
| 0 ("1cm") | 0.1525 | 0.0215 | 6.0930 | 66.1540 | 72.0481 |
| 0 ("2cm") | 0.1256 | 0.0200 | 5.2800 | 68.0773 | 82.5073 |
| 0 ("3cm") | 0.1150 | 0.0192 | 5.0035 | 64.3372 | 69.3030 |
| 0 ("4cm") | 0.1003 | 0.0172 | 4.8314 | 64.3088 | 80.1276 |
| 0 ("5cm") | 0.1006 | 0.0208 | 3.8423 | 69.5041 | 89.0593 |

***Supplemental Table 2*:** Signal-to-noise ratio values for the average RMS and the MDF performance of EMG. Note that the EMG electrodes have consistently remained attached to the skin, i.e., the testing distance of EMG did not change.
